# The Planning Fallacy in the Orthopedic Operating Room

**DOI:** 10.7759/cureus.12433

**Published:** 2021-01-02

**Authors:** Brian M Katt, Amr Tawfik, Vincent Lau, Fortunato Padua, Daniel Fletcher, Bruce Stamos, Daren Aita, Evan Conte, Arjun Saxena, Joshua Hornstein

**Affiliations:** 1 Hand Surgery, Rothman Orthopaedic Institute, Philadelphia, USA; 2 Orthopaedics, Rothman Orthopaedic Institute, Philadelphia, USA; 3 Orthopaedics, Rowan School of Osteopathic Medicine, Stratford, USA; 4 Sports Medicine, Rothman Orthopaedic Institute, Philadelphia, USA

**Keywords:** planning fallacy, operating room, efficiency, estimation, optimism bias

## Abstract

The planning fallacy posits that humans tend to underestimate the amount of time needed to complete a project and that greater complexity results in a larger difference in that estimation. If this phenomenon is present in the orthopedic operating room, it could lead to negative impacts on patients, their families, and physicians themselves. Nine fellowship-trained orthopedic surgeons at one institution were asked to give an estimate of their operative and total room times over the course of three months. Over 759 cases, the surgeons underestimated the total room times by 17.3% (p = 0.034) but did not underestimate their operative times (p = 0.590). The surgeons improved estimation of their operative time for all cases from 13.6 to 10.9 minutes of their actual time (p = 0.031) by comparing the absolute difference for the surgeons’ first 25% to the last 25% of cases. Procedures performed at the hospital underestimated operative and total room times by 8.9% and 7.4% compared to the ambulatory center, which overestimated operative times by 6.0% and underestimated total room times by 3.8% (p < 0.001). We found that the planning fallacy does exist in certain situations within the orthopedic operating room.

## Introduction

The planning fallacy, first defined by Kahneman and Tversky, is the tendency to underestimate the time needed to finish a project [1]. To demonstrate the planning fallacy, Buehler et al. found a 64% overestimation when students estimated how long it would take to finish their senior thesis [2]. The discrepancy between intention and action is mainly driven by an optimism bias. Optimism bias is a pervasive tendency to overestimate the chance of experiencing a positive event and to underestimate negative events occurring. As a whole, orthopedic surgeons are inherently optimists. In 1922, Evans quipped “no orthopedic surgeon can be successful unless he is an optimist” [3]. Although optimism is a quality that is associated with a successful orthopedic surgeon, we hypothesized it can cause surgeons to succumb to the planning fallacy with negative consequences.

This behavior, if left unchecked, could have a negative impact on patients and their families, operative room staff, and the physician. Often operating rooms will generate a daily schedule based on a surgeon’s estimated completion time for each case. The underestimation of actual case times may result in longer pre-operative wait times, poorer patient satisfaction, the need for additional hospital resources such as overtime pay and replacement staff, and surgeon burnout [4].

This study approached the planning fallacy as it pertains to the orthopedic operating room. We wondered if it existed, and, if so, was it more pronounced in certain situations. Our primary goal was to determine how accurately orthopedic surgeons’ estimate the time needed to complete operative procedures. Our secondary goals were to determine how surgeons’ accuracy of surgical time estimations correlated with increased repetition and site location of service, and how accuracy of estimations varied with increased surgical durations.

We analyzed conditions both under the control of the surgeon, with skin-to-skin time, and those dependent on the team functioning as a unit with total door-to-door time. We hypothesized that orthopedic surgeons would generally underestimate the time necessary to complete the procedure. We assumed significant variation would be present among individual surgeons. Additionally, we felt the accuracy of surgeons’ estimations would increase with increasing estimations but decrease with respect to increasing surgical duration.

## Materials and methods

Between November 2019 and February 2020, nine orthopedic surgeons fellowship-trained in foot and ankle, joint arthroplasty, sports medicine, and spine or upper extremity surgery at one institution were asked to prospectively tabulate estimated operative time (eOT), which is defined as the time from incision to completion of dressing application, and estimated total room time (eTRT), which is defined as time from patient entry into the room until exit, of their respective surgeries. Actual operative times (aOT) and actual total room times (aTRT) were collected at the time of the procedure by the nursing staff through direct measurement at the conclusion of the procedure and were recorded. Differences and absolute differences between estimated and actual operative and total room times were calculated. Surgeons manually compared the actual operative and total room times to their estimated times following their cases to self-assess the accuracy of their estimations. Self-assessment was hypothesized to help improve accuracy of surgeons’ estimations with increasing repetitions and was assessed by comparing the absolute difference for the surgeons’ first 25% to the last 25% of cases.

To test if greater surgical time resulted in a larger difference between estimated and actual times, we formed subgroups based on total room times, as tasks occurring during the non-operative time are often out of the direct control of the operating surgeon. The data were separated into groups with surgeries less than 60 minutes, between 60 and 120 minutes, and greater than 120 minutes containing 286, 346, and 127 data points, respectively. Finally, we compared estimates based on the site of service between the hospital operating rooms and ambulatory surgery centers, with 98 procedures completed at the hospital and 661 procedures completed in the ambulatory surgery center.

Statistical analysis

The overall accuracy of surgeon estimated times was assessed among the entire cohort by comparing the mean and standard deviation of estimated operative and total room times to the actual operative and total room times. Improvement of surgeon accuracy in estimating times was assessed using the mean and standard deviation of the absolute difference between the estimated and actual operative and total room times. Differences between surgical sites were assessed by comparing the mean difference between the estimated and actual operative and total room times at each site. All comparisons were conducted using two-tailed Student’s t-test, and statistical significance was defined as a p-value of less than 0.05.

Surgical complexity was assessed by comparing the mean difference between estimated and actual operative and total room times within the three subgroups described previously. Differences between subgroups were determined using one-factor ANOVA (analysis of variance). Once a statistically significant difference was determined, a Tukey-Kramer test was performed to determine between which two groups this difference existed. The p-values for these groups were then estimated using Bonferroni correction, with statistical significance defined as a p-value of less than 0.05.

## Results

Complete measurements were recorded for 759 cases, which comprised 25 foot and ankle, 88 joint arthroplasty, 298 sports medicine, 10 spine, and 338 upper extremity cases. Results of average eOTs and total room times, average actual operative times and total room times, and average absolute differences for all surgeons and each individual surgeon are summarized in Table 1.

**Table 1:** Comparison of Estimated Times to Actual Times *A p-value of <0.05 was considered statistically significant

Variable	Estimated Time	Actual Time	p-Value	Absolute Difference
	Minutes: Average ± SD			Minutes: Average (% of Actual Time)
All surgeons (n = 759)				
Operative time	56.4 ± 35.3	55.4 ± 38.0	0.590	12.3 (22.2%)
Total room time	78.2 ± 39.4	82.7 ± 44.3	0.034*	14.3 (17.3%)
Foot and ankle surgeon (n = 25)				
Operative time	79.8 ± 36.2	98.8 ± 62.4	0.195	25.0 (25.3%)
Total room time	103.6 ± 38.8	135.76 ± 68.0	0.047*	36.2 (26.7%)
Upper extremity surgeon 1 (n = 115)				
Operative time	36.0 ± 23.4	32.6 ± 28.4	0.319	10.1 (31.0%)
Total room time	44.8 ± 21.3	52.9 ± 33.1	0.023*	12.9 (24.4%)
Upper extremity surgeon 2 (n = 142)				
Operative time	30.4 ± 20.9	30.7 ± 21.7	0.889	7.9 (25.7%)
Total room time	50.4 ± 20.9	55.2 ± 25.8	0.083	10.3 (18.7%)
Upper extremity surgeon 3 (n = 81)				
Operative time	38.7 ± 27.5	42.1 ± 34.1	0.485	9.8 (23.3%)
Total room time	57.5 ± 31.3	64.0 ± 40.4	0.256	12.7 (19.8%)
Sports medicine surgeon 1 (n = 53)				
Operative time	62.9 ± 33.5	60.6 ± 38.4	0.741	17.2 (28.4%)
Total room time	93.5 ± 32.2	91.6 ± 44.1	0.798	22.6 (24.7%)
Sports medicine surgeon 2 (n = 150)				
Operative time	82.6 ± 36.7	71.0 ± 37.3	0.007*	18.1 (25.5%)
Total room time	103.5 ± 40.0	101.9 ± 42.4	0.740	15.5 (15.2%)
Sports medicine surgeon 3 (n = 95)				
Operative time	69.1 ± 26.8	69.1 ± 39.3	0.992	9.3 (13.5%)
Total room time	97.7 ± 29.2	98.0 ± 33.9	0.958	9.9 (10.1%)
Spine surgeon (n = 10)				
Operative time	132.0 ± 53.3	122.9 ± 50.8	0.701	18.3 (14.5%)
Total room time	165.0 ± 55.2	166.4 ± 56.9	0.956	13.4 (8.1%)
Joint arthroplasty surgeon (n = 88)				
Operative time	63.9 ± 11.2	72.6 ± 13.4	<0.001*	10.9 (15.0%)
Total room time	95.3 ± 10.1	104.2 ± 14.6	<0.001*	10.7 (10.3%)

The average eOT was 56.4 minutes compared to the aOT of 55.4 minutes (p = 0.590). The absolute difference was 12.3 minutes or 22.2% of the actual operative time. Absolute difference is calculated by taking the total amount of over- or underestimation as an absolute number. Therefore, on average, the study group either over- or underestimated their surgical times by 12.3 minutes. One (11%) of nine surgeons underestimated and one (11%) surgeon overestimated their operative time to reach statistical significance.

The average eTRT was 78.2 minutes compared to the aTRT of 82.7 minutes (p = 0.034). The absolute difference was 14.3 minutes or 17.3% of the actual room time. Four (44%) of nine surgeons underestimated and zero of nine surgeons overestimated their total room time to reach statistical significance.

Results of the average absolute difference between the surgeons’ first 25% cases and the last 25% of cases are summarized in Table 2. Comparing the first 25% to the last 25% of cases for all surgeons showed a decrease in the average absolute difference between the eOT and aOT from 13.6 minutes to 10.9 minutes (p = 0.031). For total room time, there was no significant difference between the eTRT of 14.0 minutes and the aTRT of 14.4 minutes (p = 0.884).

**Table 2:** Surgeon Improvement by Absolute Difference *A p-value of <0.05 was considered statistically significant

	First 25%	Last 25%	p-Value
	Minutes: Average Absolute Difference ± SD		
Operative time	13.6 ± 14.9	10.9 ± 12.6	0.031*
Total Room time	14.0 ± 16.7	14.4 ± 16.3	0.884

Results of the average difference between estimated and actual operative times and total room times for different surgical durations are summarized in Table 3. Subgroup analysis showed a statistically significant average underestimation of operative time by 8.1% in procedures longer than 120 minutes when compared to both the less than 60-minute group and the 60- to 120-minute group (p < 0.001). No significant difference existed between the less than 60-minute group and the 60- to 120-minute group (p = 0.074).

**Table 3:** Surgeon Accuracy of Time Estimation Based on Procedure Length *A p-value of <0.05 was considered statistically significant

	<60-Minute Procedure	60- to 120-Minute Procedure	>120-Minute Procedure
	Minutes: Average Difference (% of Actual Time)		
Operative time	4.1 (22.0%)	2.2 (3.5%)*	-9.3 (8.1%)*
Total room Time	1.3 (3.3%)	-3.5 (3.8%)*	-20.5 (13.5%)*
Subjects (n)	286	346	127

A statistically significant average underestimation of total room time by 13.5% existed in procedures longer than 120 minutes when compared to both the less than 60-minute group and the 60- to 120-minute group (p < 0.001). A statistically significant average underestimation of total room time by 3.8% existed in procedures between 60 and 120 minutes when compared to procedures less than 60 minutes (p < 0.001).

Results of the average difference between the estimated and actual operative times and the total room times for the two facilities are summarized in Table 4. Hospital operative times were underestimated by an average of 8.9% compared to the ambulatory surgical center, which overestimated its operative times by 6.0% (p < 0.001). The hospital underestimated its total room time by 7.4% compared to a 3.8% underestimation at the ambulatory surgical center (p < 0.001).

**Table 4:** Difference in Estimation by Facility *A p-value of <0.05 was considered statistically significant

	Hospital	Ambulatory Surgery Center	p-Value
	Minutes: Average Difference (% of Actual Time)		
Operative time	-6.92 (8.9%)	3.02 (6.0%)	<0.001*
Total room time	-8.11 (7.4%)	-2.93 (3.8%)	<0.001*
Subjects (n)	98	661	

## Discussion

The planning fallacy is a propensity to underestimate the time needed to complete a project. Kahneman stated that people assume normal operating conditions when planning. By not considering unexpected events or delays, an oversimplified and idealized approach to predicting the future occurs [5]. In our study, we hypothesized a statistically significant difference between predicted and actual case times. Our data suggest that this difference rarely occurred, but it was more common when predicting total room time.

The relatively small effect of the planning fallacy in this study may have been due to the study surgeons’ experience. Of the nine surgeons involved in our study, eight had more than 10 years in practice. Traditionally, surgeons have made estimates of their case durations based on prior experiences. It is possible that the surgeons involved in the study had a better understanding of the duration of the specific surgery due to their experience.

It is also important to consider that of the 759 cases that were involved in this study, 661 took place in an ambulatory surgical center. Prior studies have shown that patients scheduled for surgeries at ambulatory surgical centers are more likely to be younger and have lower body mass index (BMI), Charlson comorbidity index (CCI), and American Society of Anesthesiologists (ASA) scores on average than those scheduled for surgeries in the hospital [6,7]. These patient factors have been shown to be correlated to surgical duration [8]. Our facility subgroup analysis supports this, as the cases completed at the ambulatory surgical center had more accurate estimations on average than those completed in the hospital. It is possible these variables made it easier for surgeons to predict their operative and total room times when performing cases within the ambulatory surgical center.

Examining the neural mechanisms in optimism, Sharot et al. found that imagining positive events engages areas of the brain associated with emotion, memory, and prediction more intensely than imagining negative events [9]. Optimism may be an advantageous adaptation of the human brain. In order to progress forward, humans require a strong belief that they can achieve the best outcome. This belief creates motivation to pursue and accomplish set goals. Subsequently, optimists in general are recorded to work longer hours and tend to earn more as a result [10]. There are health benefits correlated with optimistic people, which include lower cardiovascular risk and all-cause mortality [11]. Optimists tend to be more motivated and more successful. While helping drive us forward, this optimistic quality does hinder the likelihood of taking precautions as well as reduce the likelihood of contemplating what can go wrong [9].

The dangers of optimism occur when underestimating negative outcomes, leading to inaccurate predictions of the future [10]. The literature provides conflicting data on the accuracy of surgeons’ estimates of operative cases. Pandit and Carey concluded that surgeons demonstrated good ability to predict actual list duration from estimates, with an overall correlation coefficient of 0.78 [12]. Dinesh and Davis-Joseph recorded estimated and actual surgical times in 753 surgical cases over a three-month period. They found that surgeons’ estimates were more accurate for shorter cases compared to longer ones [13].

In the operating room, non-operative, pre-procedure, and post-procedure steps are factors less in the surgeon’s control. These processes involve other members of the operating room team. The increase in the number of people involved creates a “coordination neglect.” Coordination neglect comes from the failure to think about the challenge of putting things together when there are more people involved. In our study, the time in the room when the surgeon is not operating was most susceptible to this neglect, with delays and miscommunications that can occur at every step of the pre- and post-procedure. For example, delays can occur in anesthesia, intubation, setting up the room, extubation, moving the patient on and off the operating table, and receiving permission to proceed to the recovery room. These processes can decrease the efficiency of the operating room. Our data support this idea, as the surgeons in our study statistically underestimated total room time. Furthermore, as the study proceeded, the surgeons were more accurate in estimating operative time but did not improve in estimating total room time. This may be due to the extraneous factors involved in the operating room that the surgeon is unable to account for.

In general, combatting the planning fallacy requires data and information. The objective would be to notice unintended consequences and ultimately be able to correct them. Akin to the data collection techniques in our study, performance tracking and data collection are the initial steps needed to overcome the planning fallacy [14]. Our study showed that when given the actual timing data to review, surgeons were able to self-correct and more accurately estimate their operative times. Inpatient operating rooms and surgery centers are resources that can collect these data for orthopedic surgeons. The data can then be used for a technique called reference class forecasting. This strategy looks at how similar projects have gone (predicted versus actual) and then adjusts the current plan accordingly. An independent person can develop a reasonable timeline and discuss with the surgeon if there is a large discrepancy between the booked time and the independent determiners of time. This party may be engaged to adjust estimated operating room times as well as perform a root-cause analysis to fix issues causing delays. This type of analysis could help orthopedic surgeons identify which aspects of the total room time they are underestimating and eventually become better at predicting the time required, reducing the negative outcomes discussed earlier.

Innovative techniques have shown success in decreasing overall room time. One example is the use of an operative table that can be brought to the side of the stretcher. The process of moving a patient from the stretcher to the operative table and back is eliminated, thus decreasing complexity and saving valuable time. This allows the added benefit of reducing potential errors during transport and less strain on the bodies of those asked to maneuver the patient [15].

Data obtained from surgeons around the country suggest that burnout rates among surgeons range from 30% to 38%. These statistics illustrate that a significant number of surgeons are experiencing personal and professional distress [16]. Continually lagging behind can create a loss of personal accomplishment, which may affect physician well-being. The constant feeling of not achieving expectations can lead to psychological distress. The fact that cases run later than estimated may prevent surgeons from having a healthy work-life balance as they are completing their work later in the day [17]. The cost of burnout may manifest as early retirement or reduction in clinical hours.

The growing emphasis on immediate gratification influences patient expectations and satisfaction. Delays in the operating room will correspondingly create tension between patients and staff. Lack of communication and information explaining the delay can moreover lead to increased anxiety for both patients and their families. These thoughts and feelings can affect their postoperative outcomes. Pre-operative anxiety has been found to be a predictive factor for increased postsurgical pain and decreased postsurgical function [18,19]. Mitigating the planning fallacy and delays within the operating room can play a part in improving both patient satisfaction and their functional outcomes.

Our study looking into the planning fallacy within the orthopedic operating room has a few key limitations. The design of the study required the participating surgeons to have knowledge of the fact that they were involved in a study. This left the surgeons susceptible to the Hawthorne effect, potentially placing more consideration into their estimates than they otherwise would in their daily planning. Furthermore, in our study, the surgeons were given information regarding the difference between their estimates and actual times. It has been demonstrated in prior studies that the planning fallacy may be due to poor recollection of how long similar events took in the past and that offering feedback improved estimates [20,21]. Providing this feedback to the participating surgeons may have minimized the presence of the planning fallacy in our study. In the future, it would be interesting to repeat the study with a group receiving feedback and another control group that did not receive any feedback to see how much of an effect giving feedback truly had in the orthopedic operating room. One final limitation of this study was the use of surgical duration as a surrogate measure for increased case complexity. In the future, it would be important to try to use different measures to stratify surgical complexity such as using the patient CCI score.

## Conclusions

This study aimed to determine if the planning fallacy was applicable to orthopedic surgeons in the operating room. As the variables and the need for coordination and case complexity increased, surgeons were less accurate in predicting total case times. Cooperation and team management may help decrease some of these discrepancies in total room time. Although there are many positive attributes of an optimism bias, it has the potential to negatively affect physician well-being, staff morale, and patient satisfaction. A recognition of this bias must be understood to effectively plan an operative day and to optimize the patient experience.
